# Anti-plaque and anti-gingivitis effect of Papain, Bromelain, Miswak and Neem containing dentifrice: A randomized controlled trial

**DOI:** 10.4317/jced.53593

**Published:** 2017-05-01

**Authors:** Abhinav Tadikonda, Kalyana-Chakravarthy Pentapati, Arun-Sreenivas Urala, Shashidhar Acharya

**Affiliations:** 1 Senior Lecturer, Public Health Dentistry, Sri Sai College of Dental Surgery, Vikarabad; 2 Associate Professor, Public Health Dentistry, Manipal College of Dental Sciences, Manipal; 3 Professor and Head, Orthodontics and Dentofacial Orthopedics, Manipal College of Dental Sciences, Manipal; 4 Professor and Head, Public Health Dentistry, Manipal College of Dental Sciences, Manipal

## Abstract

**Background:**

Patients undergoing fixed orthodontic therapy may have difficulty in maintaining a good oral hygiene due to the difficulty posed by the appliances in accessing such areas. This study aimed to compare anti-plaque and anti-gingivitis efficacy of dentifrice containing Papain, Bromelain, Miswak and Neem with a standard dentifrice among patient’s undergoing fixed orthodontic treatment.

**Material and Methods:**

Single center, single blind, parallel arm, randomized controlled clinical trial with an allocation ratio of 1:1 was conducted. Evaluation of plaque and gingivitis was done using Williams modification of Silness and Loe Plaque Index (PI) for use in orthodontic subjects and Loe and Silness’s Gingival Index (GI) at baseline and one month.

**Results:**

Inter-group comparison showed there was significantly lower mean plaque index in test (0.88 ±0.05) than in control group (1.17 ±0.05) after adjusting for the baseline plaque index (*p*<0.001). Similarly, there was significantly lower mean gingival index in test (0.87 ±0.04) than in control group (1.14 ±0.04) after adjusting for the baseline gingival index (*p*<0.001).

**Conclusions:**

The efficacy of the test dentifrice in limiting plaque and gingivitis suggests that it can be used as a home based adjunct to clinical therapy in orthodontic patients.

** Key words:**Bromelain, gingivitis, miswak, neem, papain, plaque.

## Introduction

Dental plaque is one of the chief causal agents responsible for two of the most common diseases implicated in the deterioration of oral health i.e., dental caries and gingivitis (1,2). Gingivitis if left untreated, gradually results in periodontitis (3). Plaque is described as the soft, tenacious material found on tooth surfaces which is not readily removed by rinsing with water (4).

Plaque formation begins with the formation of a glycoproteinaceous pellicle layer, constituted from components of saliva, crevicular fluid, host and bacterial cells. Though the pellicle layer primarily serves as a protective barrier, it also acts as a substrate for bacterial accumulation which invariably forms dental plaque. Prevention or removal of pellicle layer formation is the targeted approach towards limiting accumulation of plaque (5). A plethora of options ranging from professional, mechanical removal to home based chemical control of plaque exist, but patient friendly methods are practical and more favorable than clinical procedures.

The pellicle layer being protienaceous also has the propensity to absorb pigments causing extrinsic stains on teeth. Certain chemical dentifrices extensively aimed at extrinsic stain removal by altering the surface environment of the teeth which limited plaque adherence. Similarly, papain and bromelain are proteolytic enzymes which have proven to remove stains by dissolving the proteinaceous pellicle layer (6-9).

Papain and Bromelain on the basis of their proteolytic action were used in food processing as meat tenderizers and in assisting digestive process and the immune system, cancer therapy, cardiovascular function and maintenance of general health. Neem and Miswak are natural products whose extracts have proven antibacterial efficacy (10,11).

Papain a is natural enzyme, derived from latex of the Papaya fruit (Carica papaya). It is known to possess the ability to hydrolyze large proteins into smaller peptides and amino acids. Its characteristic broad substrate specificity and hydrolyzability made Papain an ideal enzymatic supplement (12).

Bromelain is extracted from the stem and fruit of Pineapple (Ananas comosus) plant. It prevents the propagation of inflammation by blocking pro-inflammatory metabolites used extensively to treat arthritis, trauma and other inflammatory processes (13).

Medically papain and bromelain in conjunction with animal proteases like trypsin and chymotrypsin offer a wide spectrum of therapeutic effects. Their collective anti-edemateous, anti-inflammatory, anti-thrombotic and fibrinolytic action has been established in laboratory and human studies. They modulate the functions of adhesion molecules on blood and endothelial cells, and also regulate and activate various immune cells and their cytokine production (14).

Neem contains isoprenoids such as nimbin, nimbinin and nimbidin which have antibacterial affects against oral streptococci and also prevent adhesion to tooth surface. They also contain Catechins that reduce oxidative burst from polymorphonuclear leukocytes thereby limiting inflammation. The antibacterial effects of Miswak are known to be mediated by the release of tannins and thiocynates that affect early colonizers in plaque such as streptococci, and the periodonto pathogen *P. gingivalis*. The thiocyanates are capable of activating the salivary peroxidase⁄ thiocyanate system, thereby exerting a potent antibacterial effect (15).

The premise that any substance disrupting the pellicle along with antibacterial agents such as neem and miswak might affect plaque formation and gingival inflammation was evaluated in our study.

Patients undergoing fixed orthodontic therapy may have difficulty in maintaining a good oral hygiene, not only due to the increased plaque accumulation around the brackets and wires but also the difficulty posed by the appliances in accessing such areas (16,17).

The aim of the following study was to compare anti-plaque and anti-gingivitis efficacy of toothpaste containing Papain, Bromelain, Miswak and Neem with a standard dentifrice among patient’s undergoing fixed orthodontic treatment. The null hypothesis was that there was no difference in the anti - plaque and anti - gingivitis effect between the test and control groups.

## Material and Methods

The study was a single center, single blind, parallel arm, randomized controlled clinical trial with an allocation ratio of 1:1. The study was approved by the Institutional Ethics Committee, Kasturba Hospital, Manipal (IEC 42/2015). The study, with possible discomforts, benefits and harms were clearly explained to the participants and informed consent was sought. The study was conducted in the clinics of the department of Orthodontics and Dentofacial Orthopaedics, Manipal College of Dental Sciences, Manipal which included systemically healthy patients, over 18 years of age, proficient in English and were undergoing fixed orthodontic therapy. Clinical prerequisites were that subjects had brackets and arch wires both in maxilla and mandible, visible plaque and gingivitis in at least 30% of the present teeth or a baseline score of 1 in plaque and gingival indices respectively and had undergone orthodontic therapy for a minimum of three months. Subjects excluded were those with multiple restorations and gross dental caries, functional or removable appliances or mini implants, under any form of topical or systemic antibiotic treatment during the past 2 weeks, current users of tobacco in any form and those allergic to the given products.

The sample size was calculated based on the formula for comparison of means. A pilot study of 8 samples with representation from both groups was conducted prior to the study to help in the determination of sample size. The type I error (α) was set at 95% and type II error (power of the study) β was set at 80%. A variance σ of 0.24 was derived by pooling the variance and obtaining the average of the Gingival Index (GI) scores of the pilot study. The expected minimal difference was set at 0.2. A sample size of 23 per group was estimated for the study and the total sample size required was 46.

All the participants were screened for inclusion and exclusion criteria and 52 participants were finally recruited. Each participant was then asked to select a numbered chip from a bowl (fish bowl method). The chips were numbered by an investigator not involved in the clinical examination or statistical analysis. Numbered opaque cardboard boxes which were pre-assigned to match with the chip number were used to allocate the dentifrices to the participants and blind the clinical examiner. Clinical examination was done by a principal investigator with the help of a recorder to assess plaque accumulation (using a disclosing solution) and gingivitis. Evaluation of plaque was done using Williams modification of Silness and Loe Plaque Index (PI) (18) for use in orthodontic subjects. Loe and Silness’s Gingival Index (GI) (19) was used to measure the gingival status. After examination all the participants were demonstrated the Charters method of tooth-brushing by the principal investigator. They were also advised to brush twice daily for 2-3 minutes using the given toothpaste and their regular toothbrush and to refrain from using any other oral health care product during the course of the study. The follow up examination was conducted after 30 days by the same investigator for plaque accumulation and gingivitis. Intra observer reliability was assessed using Intra – class correlation coefficient for the assessment of plaque and gingivitis. Intra observer reliability for plaque and gingival indices were 0.91 and 0.93 respectively.

-Interventions

Test group: Participants were given a dentifrice containing papain, bromelain, neem and miswak with 1000 ppm fluoride (commercially available as Glodent).

Control Group: Participants were given standard fluoridated dentifrice (commercially available as Colgate strong teeth).

-Statistical Analysis

All data were analyzed using SPSS version 16.0. Paired t test was used for intragroup comparison between baseline and follow up. Independent sample t test was used for intergroup comparisons. Intergroup comparisons of one month scores were done using ANCOVA after adjusting for baseline scores.

## Results

**Figure 1:**
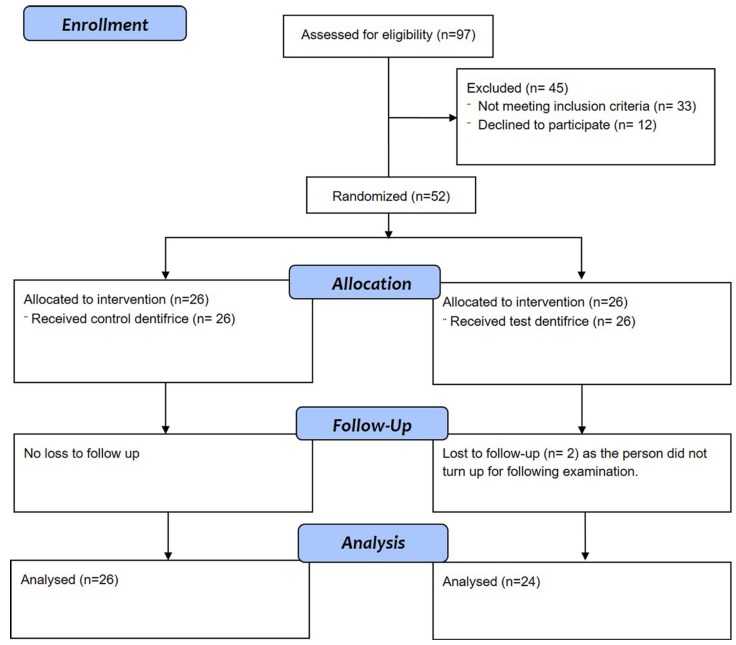
CONSORT flow chart.

A total of 97 participants were assessed for eligibility and 45 participants (33 of which did not meet the inclusion criteria and 12 of whom declined to participate) were excluded from the study. After exclusion the study group comprised of 52 participants with 26 participants in each group on whom baseline PI and GI were conducted (Fig. 1). The control group comprised of 35% (n=9) males and 65 % (17) females whereas the test group had an equal distribution of males (n=12) and females (n=12). The age range of the participants ranged from 18 to 24 years with a mean age of 20±1.72 and 20.23±1.97 in test and control groups respectively. The baseline comparison of PI and GI scores reveals no significant difference between the test and control groups ( Table 1). At the subsequent examination two participants were lost to follow up from the test group because of no show. After one month of dentifrice use there was a significant reduction in the PI and GI scores in comparison to the baseline values in both the test and control groups ( Table 1).

**Table 1:**
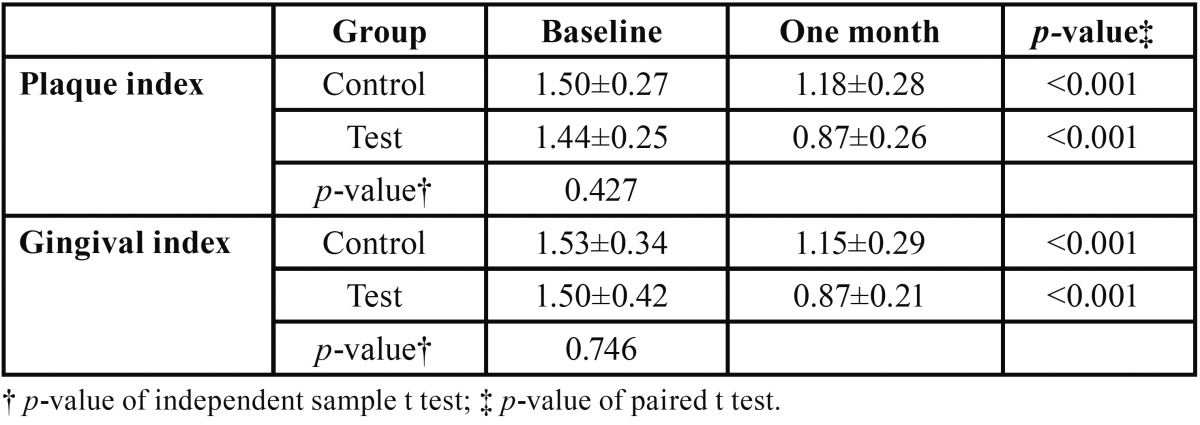
Inter-group comparison of plaque and gingival indices at baseline.

Inter-group comparison showed there was significantly lower mean plaque index in test (0.88 ±0.05) than in control group (1.17 ±0.05) after adjusting for the baseline plaque index (*p*<0.001). Similarly, there was significantly lower mean gingival index in test (0.87 ±0.04) than in control group (1.14 ±0.04) after adjusting for the baseline gingival index (*p*<0.001).

## Discussion

Our study evaluated the anti-plaque and anti-gingivitis efficacy of a dentifrice containing Papain, Bromelain, Miswak and Neem with a standard dentifrice among patient’s undergoing fixed orthodontic treatment. The retentive nature of appliances in patients undergoing fixed orthodontic therapy and difficulty in accessing certain areas severely affects the ability to maintain a good oral hygiene. Maintenance of oral hygiene in orthodontic patients can be carried out by both clinical and supplementary methods of care. While the effectiveness of the clinical procedures are inimitable the importance of adjuncts to clinical care in this patient group cannot be underscored. Thus, additional home based oral health care must be a mainstay in upholding good oral health levels. A variety of products such as mouth rinses, dentifrices, etc have been used as agents to prevent plaque accumulation and gingivitis. Dentifrices containing antimicrobial agents such as chlorhexidine, triclosan and chemical agents like sodium bicarbonate and hydrogen peroxide were also used previously. Such products are not readily available as over the counter products and have potential adverse effects on long term usage. Hence, in our study the efficacy of dentifrice containing Papain, Bromelain, Neem and Miswak in limiting plaque and gingivitis was compared with a standard readily available over the counter dentifrice.

Neem and Miswak are proven antimicrobial agents which may further enhance the properties of the dentifrice (20,21). Few short term clinical trials on Neem were conducted by Bothello *et al.* (22) and Chatterjee *et al.* (23) which showed a reduction in plaque and gingivitis comparable to Chlorhexidine gluconate. Miswak extracts in mouthwashes have also shown beneficial effects on oral health though it was restricted to improvement in gingival health and bleeding (24). Recent systematic reviews on Neem and Miswak clearly elucidate the numerous beneficial effects of these agents (20,21).

There was a significant reduction in the plaque and gingival index scores post intervention in both the groups when compared to baseline. Although there was a significant reduction in both the groups there was a significantly higher percentage reduction of plaque and gingival index scores in the test group than in the control group. This was similar to the result achieved by Pradeep *et al.*, (6) in which the test toothpaste showed a significant decrease in gingival index but not in plaque index when compared to the placebo group. This result is also in line with the studies reported by Kalyana *et al.*, (7) and Patil *et al.*, (9) in which the ability of these proteolytic dentifrices was demonstrated in the removal of extrinsic stains.

The single blinded nature was one of the disadvantages of the study. But, the stark difference in the tastes of the dentifrices precluded the blinding of the participants. Few participants didn’t maintain adequate oral hygiene measures before the enrolment due to which there was gingival over growth. Also, some participants had additional orthodontic attachments (modules, hooks and buttons). In these participants there was difficulty in the assessment of the plaque as there was hardly any tooth surface. All the participants were asked if there were any issues causing discomfort in the usage of the products to which none had any complaints deeming the test dentifrice acceptable.

The efficacy of the dentifrice in limiting plaque and gingivitis suggests that it can be used as a home based adjunct to clinical therapy in orthodontic patients.
